# B7-H3 is Overexpressed in Patients Suffering Osteosarcoma and Associated with Tumor Aggressiveness and Metastasis

**DOI:** 10.1371/journal.pone.0070689

**Published:** 2013-08-05

**Authors:** Ling Wang, Qi Zhang, Wei Chen, Baoen Shan, Yang Ding, Guochuan Zhang, Nana Cao, Lei Liu, Yingze Zhang

**Affiliations:** 1 Hebei Bone Research Institute, Third Hospital of Hebei Medical University, Shijiazhuang, Hebei, P.R. China; 2 Hebei Cancer Research Institute, Fourth Hospital of Hebei Medical University, Shijiazhuang, Hebei, P.R. China; 3 Department of Orthopaedic Surgery, Third Hospital of Hebei Medical University, Shijiazhuang, Hebei, P.R. China; 4 Department of Pathology, Third Hospital of Hebei Medical University, Shijiazhuang, Hebei, P.R. China; 5 Department of Orthopedic Oncology, Third Hospital of Hebei Medical University, Shijiazhuang, Hebei, P.R. China; Inserm U606 and University Paris Diderot, France

## Abstract

B7-H3 is a member of the B7-family of co-stimulatory molecules, which has been shown to be broadly expressed in various tumor tissues, and which plays an important role in adaptive immune responses. The role of B7-H3 in osteosarcoma, however, remains unknown. In this study we used immunohistochemistry to analyze B7-H3 expression in 61 primary osteosarcoma tissues with case-matched adjacent normal tissues, and 37 osteochondroma and 20 bone fibrous dysplasia tissues. B7-H3 expression was expressed in 91.8% (56/61) of the osteosarcoma lesions, and the intensity of B7-H3 expression in osteosarcoma was significantly increased compared with adjacent normal tissues, osteochondroma and bone fibrous dysplasia tissues (*p*<0.001). Patients with high tumor B7-H3 levels had a significantly shorter survival time and recurrence time than patients with low tumor B7-H3 levels (*p*<0.001). Moreover, tumor B7-H3 expression inversely correlated with the number of tumor-infiltrating CD8^+^ T cells (*p*<0.05). In vitro, increasing expression of B7-H3 promotes osteosarcoma cell invasion, at least in part by upregulating matrix metalloproteinase-2 (MMP-2). In conclusion, our study provides the first evidence of B7-H3 expression in osteosarcoma cells as a potential mechanism controlling tumor immunity and invasive malignancy, and which is correlated with patients’ survival and metastasis.

## Introduction

Osteosarcoma is the most common primary malignant bone tumor and predominantly affects adolescents and children [1], [2]. Despite dramatic advances in wide-margin surgery and intensification of chemotherapeutic treatment, the 5-year disease-free survival and overall survival rates have reached a plateau at about 50–60% [3], [4]. Osteosarcoma has a propensity for local invasion and early lung metastasis, which results in only 20% metastatic patients surviving during the first 5 years [5], [6]. Due to the development of multiple types of chemoresistance, better prognostic factors and more effective therapeutic modalities are badly needed for patients with refractory osteosarcoma.

Adoptive cellular immunotherapy is currently accepted as a suitable alternative to surgery and chemotherapy for osteosarcoma patients because of its easy and painless administration and improved safety [7], [8]. It is well known that optimal activation of antigen-specific lymphocytes requires a combination of T-cell receptors (TCRs) and costimulatory signals [9]. In vivo manipulations of the T-cell costimulatory pathway are also being explored as a means to evoke immune responses for treatment of osteosarcoma [10]. In addition to the traditional B7-1 and B7-2 family members, other B7-CD28 family members have been discovered, including B7-H1 [11], B7-H2 [12], B7-H3 [13], B7-H4 [14], B7-DC [15] and B7-H6 [16]. Among these, B7-H3 is a currently controversial costimulatory molecule, which plays crucial roles after initial antigen priming in cooperation with a putative counterreceptor. B7-H3 protein expression has been described in numerous human malignancies of the lung, stomach, breast, prostate and other tissues [17]–[20]. Related to this, aberrant tumor cell B7-H3 expression has recently emerged as a possible mechanism whereby human tumors might escape host immune surveillance.

Despite these studies, the association of B7-H3 expression with clinical outcome in patients with osteosarcoma has not yet been investigated. In the present study, we profiled B7-H3 protein expression in tumor specimens of osteosarcoma, and analyzed the relationship between this expression and clinicopathological variables. Furthermore, we also examined the association between B7-H3 expression in tumor cells and tumor-infiltrating lymphocytes (TILs). These data are the first to demonstrate B7-H3 expression in osteosarcoma lesions and, more remarkably, suggest that B7-H3 may be clinically relevant in osteosarcoma, potentially facilitating tumor progression by undermining host immunity. Moreover, we carried out functional experiments to further elucidate the possible underlying cellular functions of B7-H3 affecting osteosarcoma malignancy. Together, these data suggest that B7-H3, a coinhibitory T-cell regulator, may represent a novel prognostic and invasive marker, as well as a potential target for alternative immunotherapy approaches in patients with osteosarcoma.

## Materials and Methods

### Cell Lines

Three human osteosarcoma cell lines (U-2OS, MG-63, Saos-2) were obtained from the Cell Bank of the Chinese Academy of Sciences (Shanghai, China) and cultured according to the instructions from American Type Culture Collection (ATCC). Cell lines were all maintained in suitable medium supplemented with 10% fetal bovine serum and 1% penicillin/streptomycin.

### Patients

In this study, a total of 118 paraffin-embedded specimens, including 61 osteosarcoma, 37 osteochondroma and 20 bone fibrous dysplasia patients, from the department of the division of surgical pathology, the third hospital of Hebei Medical University respectively from 2004–2009, with complete histopathology and follow-up information. None of the patients received pre-operative chemotherapy or radiotherapy before surgery. All patients provided written informed consent for tissue sample analysis. The study protocol was approved by The Institutional Ethics Committee at Third Hospital of Hebei Medical University. We did not conduct research outside our country of residence. All participants provide their written informed consent to participate in this study. Our ethics committees approved this consent procedure. Each individual in this manuscript has given written informed consent to publish these case details.

Survival periods were counted in months from the date of first visit to date of death or last follow-up before study closure. 61 osteosarcoma patients had a median follow-up 5 years (ranging from 2 to 7.3 yeras), and these data were used in survival analyses. Besides, 20 patients had metastasis at the first visit (synchronous), 13 patients developed metastasis during follow up (metachronous) and 28 were metastasis-free. Metastases were normally localized in lung and the primary sites were in extremity bones.

### Immunohistochemical Staining

Immunohistochemistry was performed using the Dako EnVision™ method according to the manufacturer’s instructions. In brief, 4-µm thick consecutive sections were cut by microtome, dewaxed in xylene and rehydrated through graded ethanol solutions. Antigens were retrieved by heating the tissue sections at 100°C for 30 min in EDTA solution. Sections were cooled down and immersed in 0.3% H_2_O_2_ solution for 20 min to block endogenous peroxidase activity, and then rinsed in PBS for 5 min, blocked with 5% BSA at room temperature for 20 min, and incubated with primary antibodies against CD4, CD8 (diluted in 1∶100) or B7-H3 (final concentration in use, 5 µg/ml) at 4°C overnight. Positive controls were from immersion fixed paraffin-embedded sections of human melanoma. Negative controls were performed by replacing the specific primary antibody with PBS (figure S1). After three PBS washes, sections were incubated with secondary antibodies for 30 min at room temperature. Diaminobenzene was used as the chromogen and hematoxylin as the nuclear counterstain. Sections were dehydrated, cleared and mounted.

### Evaluation of Immunohistochemical Staining

Evaluation of B7-H3 staining in cancer cells was evaluated by authorized pathologists who had no knowledge of the patients’ clinical status and outcome. B7-H3 expression scores were given separately for the stained area and for the intensity of staining. Quantification was made as follows; ≤33% of the cancer cells: 1, >33 to ≤66% of the cancer cells: 2, >66% of the cancer cells: 3; intensity of staining: absent/weak: 1, moderate: 2, strong: 3. The intensity of B7-H3 staining was considered weak when either cytoplasmic expression or rare membranous condensation was present, moderate when incomplete and discontinuous moderate membranous expression was present, and strong when complete membranous expression of the molecule was present. Each section had a final grade that derived from the multiplication of the area and intensity scores. Sections with a final score of ≤3 were classified as tumors with low B7-H3 expression, whereas sections with a final score of >3 were classified as tumors with high B7-H3 expression.

The prevalence of CD4^+^ or CD8^+^ T cells was semi-quantitatively evaluated at high power magnification (×400) according to the number of each kind of cells per areas. Areas with 0–5 CD4^+^ or CD8^+^ cells were recorded as (0), areas with 6–15 CD4^+^ or CD8^+^ cells were recorded as (1), and areas with >15 CD4^+^ or CD8^+^ cells were recorded as (2). Tumor samples were examined by two observers in a blind manner. 0 and 1 was classified as low T cell infiltration, while 2 was classified as high T cell infiltration. The individuals were grouped and compared according to B7-H3 expression.

### Induction of B7-H3 in Cultured Osteosarcoma Cells and Analysis

To determine the effects of IFN-γ, IL-4, and transforming growth factor (TGF)-β1 on expression of B7-H3 in cultured osteosarcoma cells and incubated with 40 ng/ml of recombinant IFN-γ, IL-4, and TGF-β1. After 24 and 48 h of stimulation, cells were harvested and reverse transcription-polymerase chain reaction (RT-PCR) and western blotting were employed to measure the mRNA and protein expression of B7-H3.

### Transient Transfection of B7-H3 Overexpressing or Sicilencing Plasmid

To further analyze the role of B7-H3 in osteosarcoma malignancy, we transfected Saos-2 cells with the B7-H3 cDNA plasmid using Lipofectamine^2000^ (Invitrogen, CA). The mammalian expression vector pCMV6-AC-GFP (Origene Technologies, Inc.) was used for overexpression of *B7-H3* gene (NM_001024736). The plasmid has been derived from single colony E.coli cultures and purified through OriGene’s ion-exchange purification system (PowerPrep. HP Midiprep Kits with Prefilters NP 100024). The empty vector plasmid pCMV6-AC-GFP was also purchased from OriGene’s company and used as control. Next, we transfected osteosarcoma MG-63 cells with the *B7-H3* shRNA plasmid to target B7-H3 expression. Each shRNA vector is cloned in pGFP-V-RS plasmid (Origene Technologies, Inc.) under U6 promoter for mammalian cell expression. The set sequence of the B7-H3 shRNA contains 5 vials of gene-specific shRNA expression vectors in pGFP-V-RS plasmid. We selected the most efficient one to carry out the following experiment. This sequence of the *B7-H3* shRNA is 5′-TGAAACACTCTGACAGCAAAGAAGATGAT-3′. The plasmid containing a non-effective shRNA cassette against green fluorescent protein as a scrambled negative control. In brief, about 3×10^5^ cells were seeded per well in a 6 well plate. After 24 h, the cells were transfected with 1.5 µg of cDNA or shRNA plasmid for 6 h, and the media were replaced with fresh growth medium. At 48 h after transfection, cells were harvested for analysis.

### RT-PCR

Total cellular RNA was extracted for RT-PCR as described previously. Primers included were the following: B7-H3 (sense: 5′-ctctccaaaggaaagcgaggtggacat-3′, antisense: 5′-agactgtacactgtaggtgctgaaatca-3′), β-actin (sense: 5′-atgggtcagaaggattcctatgt-3′; antisense: 5′-tcaggaggagcaatgatcttga-3′). PCR was programmed as follows: 94°C for 2 minutes, 30 cycles of 94°C for 30 seconds, 55°C for 30 seconds, 72°C for 30 seconds, 72°C for 10 minutes, hold at 4°C. RT-PCR products were analyzed via 1.5% agarose gel electrophoresis and stained with ethidium bromide for visualization using ultraviolet light.

### Western Blotting Analysis

Protein from osteosarcoma cells were extracted for western blotting as described previously [21]. The primary antibodies used included antibodies to B7-H3 (Abcam, CA), MMP-2 (Santa Cruz Biotechnology) and GAPDH (Cell Signaling Technology).

### Invasion Assay

Osteosarcoma cells were harvested with 0.05% trypsin containing 0.02% EDTA (Sigma-Aldrich), and suspended in α-MEM at a concentration of 5×10^4^ cells/well. Invasion assay was conducted as described previously [22]. All experiments were performed in triplicate.

### Statistical Analysis

Results are reported as mean ± SD. All the experimental data were analyzed by the SPSS 17.0 statistical software package. The Mann-Whitney U test, χ2 test, Pearson chi square test or Spearman rho test were performed for comparative statistical evaluations among groups and for correlation analysis with histological and clinical parameters (age, gender, tumor stage, tumor grade, and postoperative survival). Survival periods were counted in months from the date of first visit to date of death or last follow-up before study closure. We used Kaplan-Meier method to estimate the overall survival for low and high levels of B7-H3 expression. A *p* value<0.05 was considered as statistically significant.

## Results

### B7-H3 Overexpression and Associated with Clinical Features in Osteosarcoma Tissues

**Figure 1:**
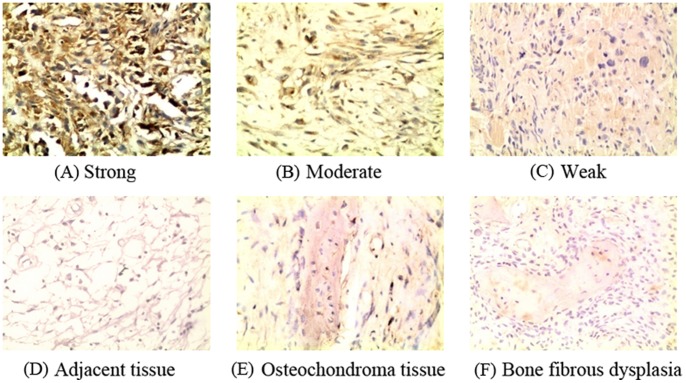
Representative immunostaining for B7-H3 expression in osteosarcoma, osteochondroma and bone fibrous dysplasia tissue. B7-H3 immunostaining in osteosarcoma tissues (A) strong positive, (B) moderate positive, (C) weak positive. B7-H3 immunostaining in osteosarcoma adjacent tissues (D), osteochondroma tissues (E) and fibrous dysplasia tissues (F). ×200 magnification.

Among all osteosarcoma patients under study, B7-H3 was highly expressed, with a median of 90% of tumor cells staining positive. Only five (8.2%) specimens did not have evidence of tumor cell expression of B7-H3. Immunostaining results showed that the immunolocalization of B7-H3 molecule was predominantly in the membrane and cytoplasm of tumor cells. According to the staining intensity, there were nine (16.1%) cases with weak tumor B7-H3 intensity, 29 (51.8%) with moderate intensity, and with 18 (32.1%) marked intensity. Depending on the area of positive immunoreactivity, a final overall score (high or low tumor B7-H3 expression) was established as described in the Methods section. A total of 60.7% of tumor samples were identified as high B7-H3 staining, while 39.3% showed a lower degree of B7-H3 staining. The case-matched adjacent normal tissues were essentially negative for B7-H3 staining. In the osteochondroma and bone fibrous dysplasia tissues, B7-H3 expression was detected in 21 (56.8%) and 18 (85%) of these specimens, respectively. In osteochondroma tissue, B7-H3 expression was weak in 14 (66.7%) cases, with seven (33.3%) cases showing moderate intensity. Although almost all bone fibrous dysplasia tissues reacted positively to B7-H3 antibody, immunostaining results showed faint and diffuse membrane staining in these samples. Unsurprisingly, the level of B7-H3 expression was significantly increased in osteosarcoma compared with adjancent normal tissues, osteochondroma and bone fibrous dysplasia tissues (*p*<0.001, Wilcoxon W test). Representative immunostaining images of B7-H3 are presented in Figure 1.

Pertinent clinicopathological findings of the enrolled patients are summarized in Table 1. We evaluated the correlation of the B7-H3 expression with various clinicopathological data. Although B7-H3 was expressed in osteosarcoma across all tumor stages, tumors with high B7-H3 expression had more advanced Ennecking stage (*p* = 0.037) and common pulmonary metastasis (*p* = 0.008). However, B7-H3 expression was not associated with age (*p* = 0.747), gender (*p* = 0.090), lesion site (*p* = 0.219), histological subgroups (*p* = 0.819) or differentiation status (*p* = 0.079). These data suggested that B7-H3 expression might be functionally important in tumor progression and metastasis in osteosarcoma.

**Table 1:** Relationship between B7-H3 expression on tumor cells and clinicopathological features.

Clinical features	Number of cases	B7-H3 expression		
		Low	High	*p*-value
Age				
≤20	34	14	20	0.747
>20	27	10	17	
Gender				
Male	35	17	18	0.090
Female	26	7	19	
Site				
Femur	30	13	17	0.219
Tibia	18	9	9	
Others	13	2	11	
Ennecking stage				
I	6	5	1	**0.037**
II	40	15	25	
III	15	4	11	
Histologic type				
Osteoblastic	35	14	21	0.819
Chondroblastic	14	4	10	
Fibroblastic	7	3	4	
Others	5	3	2	
Differentiation status				
High	38	20	23	0.079
Low	18	4	14	
Pulmonary metastasis				
Yes	33	8	25	**0.008**
No	28	16	12	

Values in bold signify *p*<0.05.

### B7-H3 Expression Inversely Correlated with the Number of CD8^+^ T Cells in Human Osteosarcoma Tissues

In several independent studies, B7-H3 has been shown to play an immunosuppressive role in human malignant tumor cells, and is correlated with the number of TILs, as well as being associated with increased disease severity [23], [24]. We next examined the intensity of TILs infiltration in specimens from all 61 osteosarcoma patients, sorted by CD4 and CD8 immunostaining. No significant difference was found in the intensity of CD4^+^ T cell infiltration between B7-H3 high-expressing and low-expressing tumors (*p* = 0.299). However, B7-H3 expression in tumor sections was inversely correlated with the density of infiltrating CD8^+^ T lymphocytes (*p* = 0.004) (Table 2). This result supports the idea that B7-H3 may play an important role in suppressing immune surveillance of osteosarcoma.

**Table 2:** Correlation between infiltrating T lymphocytes and B7-H3 expression in osteosarcoma tissues.

InfiltratingT lymphocytes	B7-H3 expression number of cases				
CD4+	Low	Ratios (%)	High	Ratios (%)	*p*-value
Low infiltrating	11	47.8	12	52.2	0.299
High infiltrating	13	34.2	25	65.8	
CD8+					
Low infiltrating	9	25.0	27	75.0	**0.005**
High infiltrating	15	60.0	10	40.0	

Values in bold signify *p*<0.05.

### B7-H3 Expression Correlated with Poor Prognosis and Metastasis-free Survival in Osteosarcoma Patients

**Figure 2:**
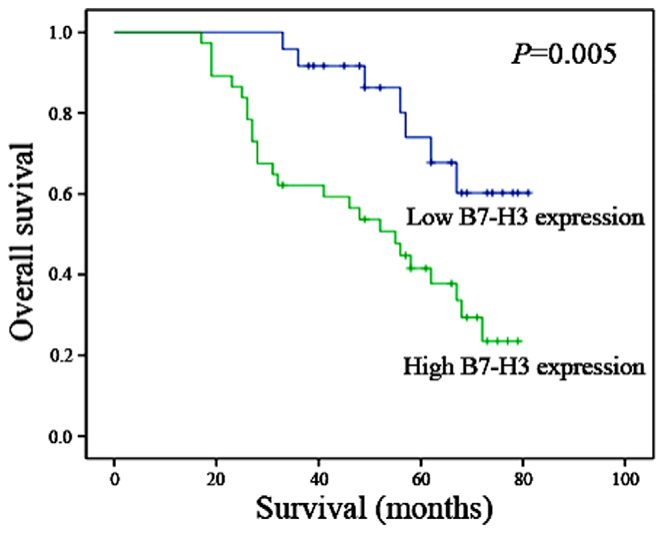
Overall survival of 61 osteosarcoma patients in relation with B7-H3 protein expression. Cumulative survival time was calculated using the Kaplan-Meier method and analyzed using the log-rank test. Patients with high tumor B7-H3 protein expression had a significantly poorer prognosis than patients without or with low tumor B7-H3 protein expression (*p* = 0.005). The median survival of patients with high tumor B7-H3 protein expression was 47.1 months in contrast to 58.3 months in patients with low tumor B7-H3 protein expression.

We next analyzed whether there was an association between survival and B7-H3 expression in osteosarcoma. Cumulative survival time was calculated using the Kaplan-Meier method and analyzed using the log-rank test. We found that carcinoma patients with high B7-H3 expression had significantly shorter survival times (*p* = 0.011, Figure 2): only 17 (58.6%) of carcinoma patients with high B7-H3 expression were alive at the time of study, compared with 12 (41.4%) carcinoma patients with low B7-H3 expression after diagnosis (*p* = 0.003). Moreover, the average time to recurrence was 38.8 months for patients with low B7-H3 expression, compared to 25.7 months for patients with high B7-H3 expression (F = 1.344, t = 2.601, df = 35, *p* = 0.014). Univariate analysis demonstrated that patients with tumors expressing high levels of B7-H3 were more likely to experience cancer recurrence and death compared with patients with tumors with low B7-H3 expression. Of the 33 metastatic patients, 75.8% were positive for B7-H3 (*p* = 0.009). In summary, differential B7-H3 expression status in tumor is associated with poor survival in high tumor stage and metastatic osteosarcoma patients.

### Cultured Osteosarcoma Cell Lines MG-63, U-2OS and Saos-2 Constitutively Express B7-H3

**Figure 3:**
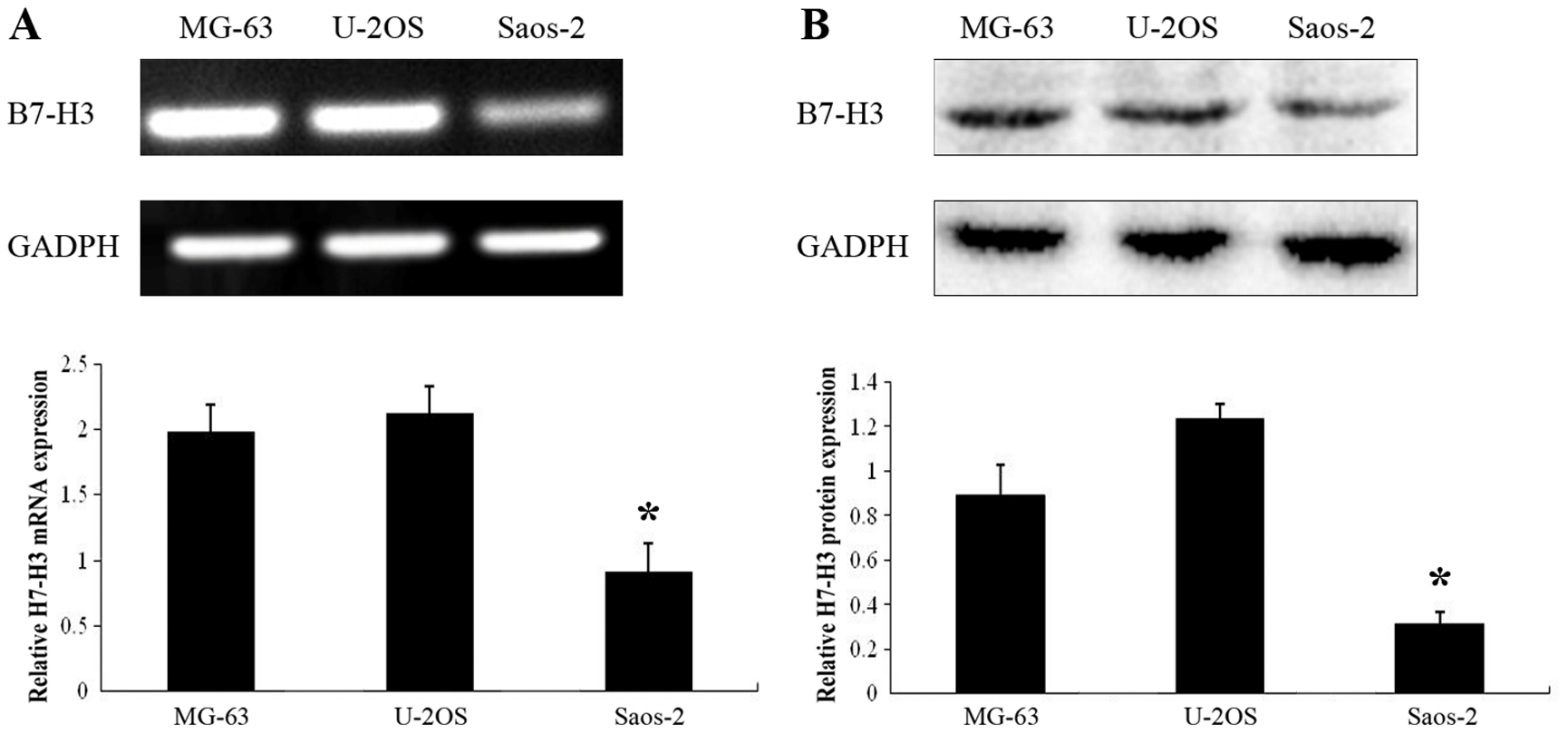
Constitutive gene expression of B7-H3 in three osteosarcoma cell lines. (A) validation of B7-H3 mRNA level in osteosarcoma cells with RT-PCR analysis. GAPDH was used as an internal control. (B) validation of B7-H3 expression in osteosarcoma cells with western blot analysis. GAPDH was used as an internal control. Histogram represents densitometric analysis of the ratio of B7-H3 and GAPDH bands. Experiments were repeated at least 3 times and the mean value was calculated. *p<0.05, compared with the other two kinds of cells. *p* values were determined by one-way ANOVA.

To test whether osteosarcoma cells express B7-H3 in vitro, we performed RT-PCR and western blotting. All tested cultured osteosarcoma cell lines constitutively expressed B7-H3 mRNA and protein at different levels under normal conditions (Figure 3). Saos-2 cells exhibited the lowest *B7-H3* gene expression compared with the other two cell lines (*p*<0.05). Although U-2OS cells contained slightly higher *B7-H3* gene expression compared with MG-63 cells, the differences did not reach statistical significance.

### IFN-γ Markedly Increased B7-H3 Expression in Osteosarcoma Cells

**Figure 4:**
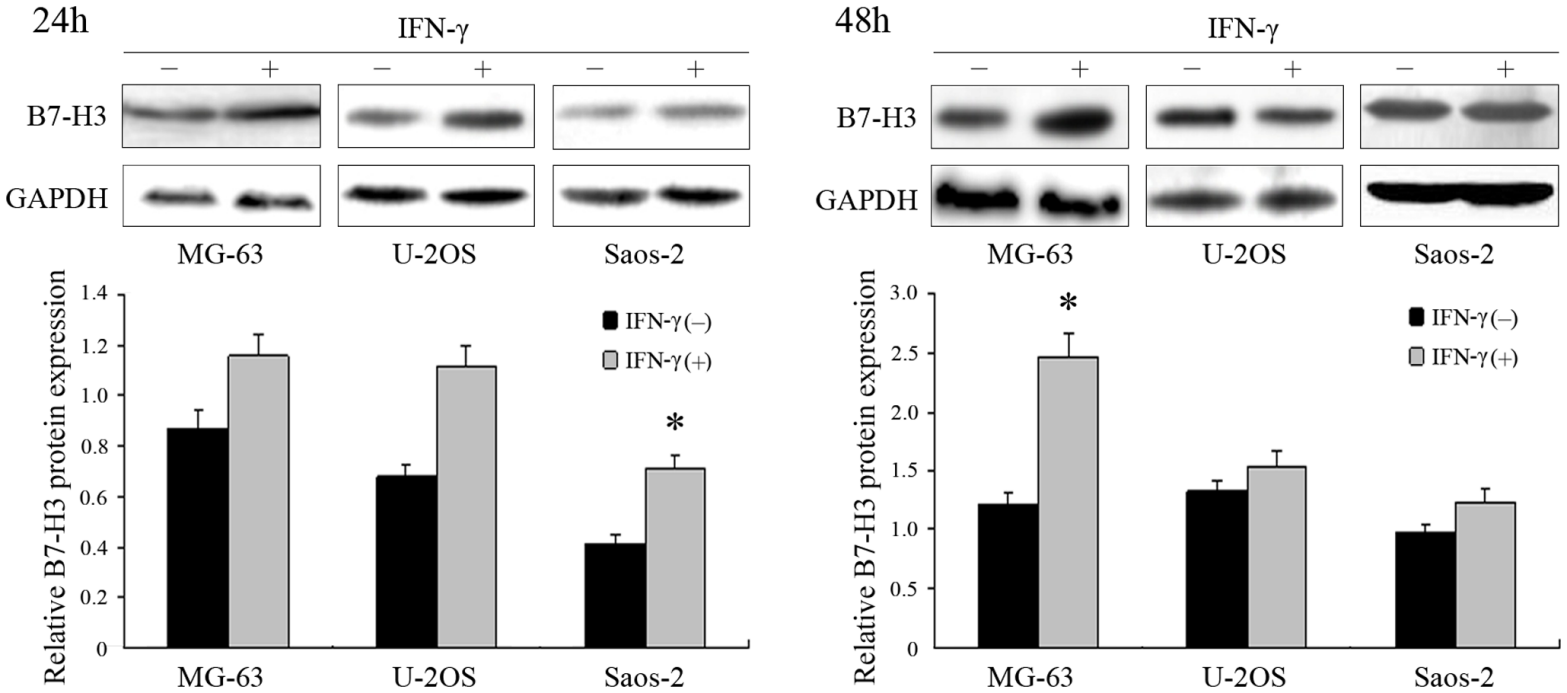
Effects of treatment with IFN-γ on MG-63, U-2OS and Saos-2 osteosarcoma cells with western blot analysis. GAPDH was used as an internal control. Histogram represents densitometric analysis of the ratio of B7-H3 and GAPDH bands. Experiments were repeated at least 3 times and the mean value was calculated. *p<0.05 denotes significant differences between osteosarcoma cells and those treated with IFN-γ. *p* values were determined by Paired Student’s t test.

Treatment with 40 ng/ml recombinant IFN-**γ** markedly increased the expression of B7-H3 in MG-63 (1.33-fold), U-2OS (1.65-fold) and Saos-2 cells (1.73-fold) after 24 h (Figure 4A). In U-2OS and Saos-2 cells, the effect induced by IFN-**γ** treatment became faint after 48 h, whereas in MG-63 cells, IFN-**γ** treatment resulted in a further increase in B7-H3 expression at 48 h (2.03-fold) and almost disappeared after 72 h (Figure 4B). However, IL-4 or TGF-β1 treatment induced no significant change in B7-H3 expression in the above three cell lines after 24 or 48 h.

### Increasing Expression of B7-H3 Promotes Osteosarcoma Cell Invasion in vitro

**Figure 5:**
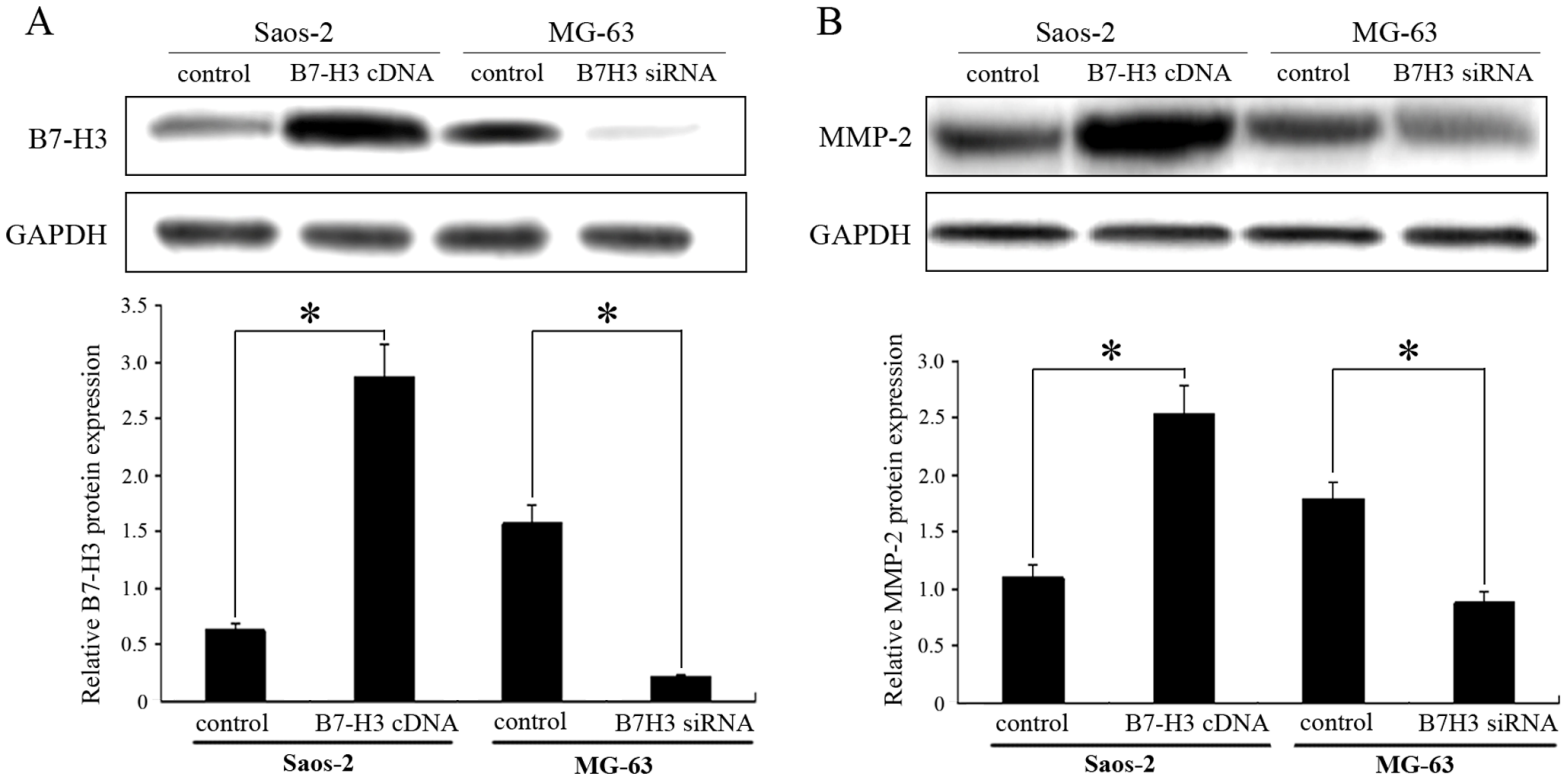
Overexpression or silencing of B7-H3 expression regulates the expression of MMP-2 in osteosarcoma cells with western blot analysis. GAPDH was used as an internal control. Histogram represents densitometric analysis of the ratio of B7-H3, MMP-2 and GAPDH bands. Experiments were repeated at least 3 times and the mean value was calculated. *p<0.05, compared with control cells. *p* values were determined by Paired Student’s t test.

**Figure 6:**
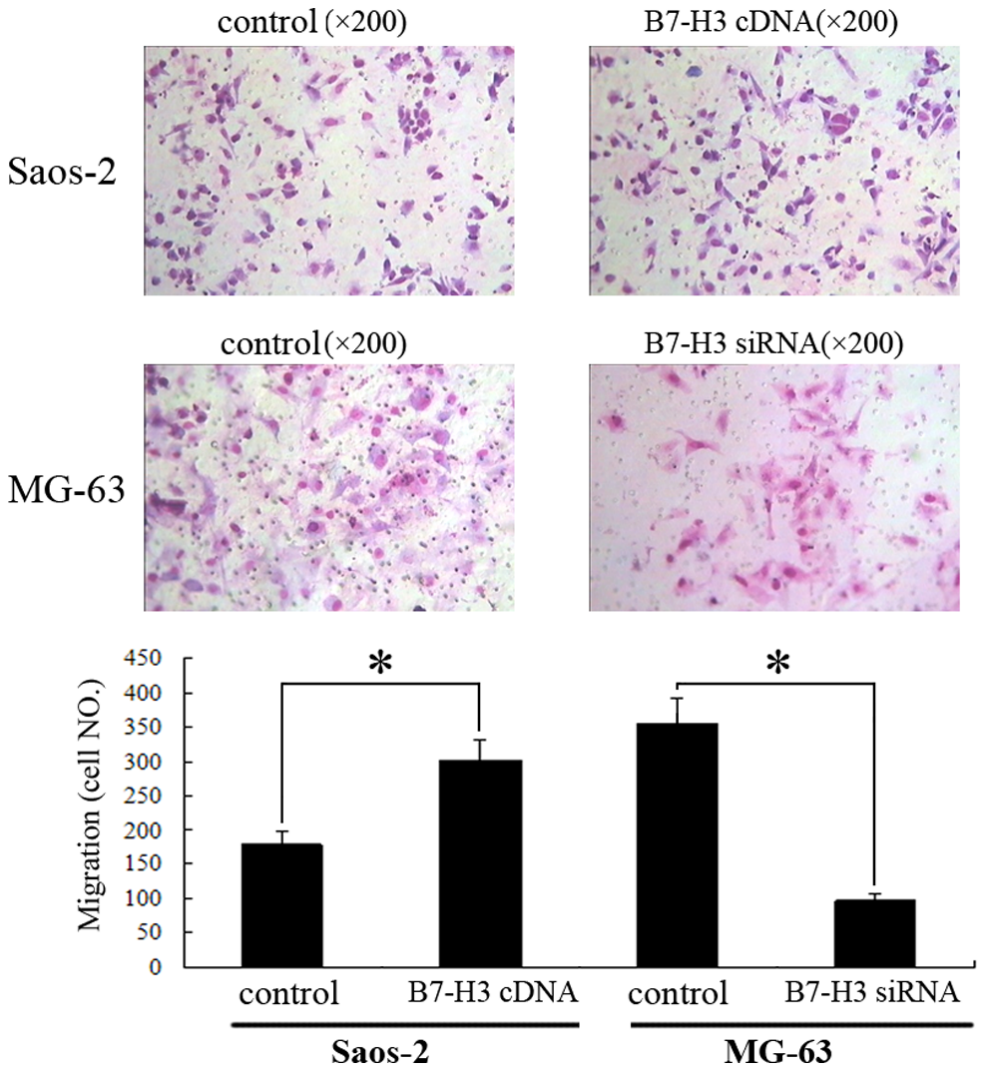
Overexpression or silencing of B7-H3 expression enhances or suppresses invasive ability of osteosarcoma cells in vitro. *p<0.05, compared with control cells. *p* values were determined by Paired Student’s t test.

Next, we used different approaches (B7-H3 cDNA or siRNA transfection) to increase or decrease B7-H3 expression to determine whether upregulation of B7-H3 enhances osteosarcoma cell malignancy. After B7-H3 cDNA transfection in Saos-2 cells, B7-H3 protein expression was upregulated significantly after 48 h (Figure 5A). B7-H3 overexpressing Saos-2 cells also exhibited markedly increased ability of invasion, compared with the vector controls, as assayed by transwell invasion chamber (Figure 6). Our data suggest therefore that increasing B7-H3 expression increases invasion in osteosarcoma cells. Additional evidence for this effect emerged from experiments in which knockdown of B7-H3 expression attenuated osteosarcoma cell invasion in MG-63 cells (Figure 6).

Previous studies have suggested a link between the increased expression of several MMPs, such as MMP-2, and osteosarcoma invasiveness, leading us to investigate the association between B7-H3 and MMP-2 [25]. We found that MMP-2 protein levels were increased in B7-H3-transfected osteosarcoma cells, while the expression of MMP-2 was decreased after B7-H3 siRNA transfection in MG-63 cells compared with the vector controls (Figure 5B).

## Discussion

Osteosarcomas are the most common primary malignant tumors of bone, generally following an aggressive clinical course, and represent a major therapeutic challenge [26]. Despite the infiltration of large numbers of immune cells into the osteosarcoma, the immune system fails to prevent disease development and progression. B7-H3 is another member of the B7 family molecules, which serves as an accessory costimulatory regulator of T cell responses following initial T cell priming. The exact physiological function of B7-H3 and, especially, its role in the development and progression of human osteosarcoma remain underdefined.

In the present study, immunohistochemistry results showed that B7-H3 expression is significantly upregulated in primary tumor lesions of osteosarcoma in comparison to osteochondroma and bone fibrous dysplasia tissues. In addition, there is a correlation between the level of B7-H3 expression and clinical data, such as tumor Ennecking stage and metastasis status. Furthermore, it is noteworthy that tumor cell-associated B7-H3 expression significantly correlated with poor postoperative survival. Therefore, we speculate that tumor-associated B7-H3 expression might act as a negative regulator of antitumor response in osteosarcoma. Our findings are in accordance with previous reports on the negative function of B7-H3 in tumor immunity. Initially, two independent research groups reported that human and murine B7-H3 could inhibit CD4^+^ T cell activation and the production of effector cytokines [27]. Further data in support of a coinhibitory role of B7-H3 in the regulation of immune response came from in vivo experiments showing that Th1-mediated hypersensitivity, and that the onset of experimental autoimmune encephalomyelitis and allergic conjunctivitis were augmented in B7-H3 knock-out mice [28], [29]. Moreover, B7-H3 has also been implicated as a potential coinhibitor of antitumor immunity. For example, several independent studies have shown that markedly increased expression of B7-H3 protein in human malignant tumor cells is associated with increased disease severity in breast cancer [19], colorectal carcinoma [30], hepatocellular carcinoma [31], prostate cancer [32], non-small-cell lung cancer [33] and neuroblastoma [34].

In our study, we have also demonstrated that B7-H3 expression is negatively associated with the intensity of infiltrating CD8^+^ T lymphocytes in tumor sites, suggesting that one of the most important contributions of B7-H3 expression in this malignancy is the impairment of host T cell-mediated immunity by negatively regulating T lymphocyte infiltration. Decreased numbers of tumor-infiltrating immune cells, including T, B and natural killer (NK) cells, have been shown to correlate with decreased survival times in human osteosarcoma [35], [36]. Moreover, a recent study has shown that the ratio of CD8^+^ T cells to regulatory T cells is inversely associated with the outcome of osteosarcoma in dogs [37]. B7-H3 may inhibit the infiltration of CD8^+^ T cells into tumor tissues, and might also suppress tumor immunogenicity by inhibition of tumor infiltrating CD8^+^ T cells. Although these results strongly implicate a tumor-suppressive role of B7-H3, the exact physiological and pathological role of B7-H3 remains elusive, since B7-H3 has also been shown to stimulate proliferation and cytokine production of both CD4^+^ and CD8^+^ T-cells [13]. In tumor immunity, evidence of a possible tumor protective effect of B7-H3 expression comes from clinical investigation of the expression of B7-H3 in human gastric [38] and pancreatic [39] carcinoma. The reason for the seemingly contradictory effects of B7-H3 in cancer might be attributed to varying counter-receptors involved in different tumor entities. Depending on the affinity of differential receptors, tumor-associated B7-H3 may have distinct functional effects on receptor-bearing cells. Another possible interpretation is that cancers may express aberrant forms of B7-H3 on the cancer cell surface, resulting in a highly diverse pattern of B7-H3 interacting with different tumor cells.

Osteosarcoma is an aggressive malignant bone disorder and is one of the leading causes of cancer-related death worldwide due to metastasis. In our study, our clinical results showed that high B7-H3 expression is a significant and persistent predictor of tumor metastasis. Based on these results, we speculate that MG-63 cells might contain the highest level of B7-H3, U-2OS cells express moderate levels, and Saos-2 cells contain the lowest expression level. However, RT-PCR and western blotting results showed that B7-H3 was overexpressed in U-2OS and MG-63 cells, with slightly higher levels in the former. This may be attributed to the U-2OS cell line having more fibroblastic than osteobastic characteristics [40]. As for the other two cell lines, B7-H3 was significantly higher in MG-63 cells than in Saos-2 cells. Moreover, we demonstrated that upregulation or downregulation of B7-H3 could promote or inhibit osteosarcoma cell invasion in vitro, respectively. Osteosarcoma is an aggressive malignant bone tumor and is highly associated with expression of the matrix metalloproteinases (MMPs) [25]. Of the MMPs, MMP-2 is present in large quantities in cancer tissues, including human osteosarcoma, and accumulating evidence indicates that MMP-2 plays a critical role during tumor invasion and metastasis [41]. However, the mechanism of action between B7-H3 and MMP-2 is still unknown. In our study, we found that B7-H3 regulated invasion of osteosarcoma cells at least partly through MMP-2. Similar conclusions were drawn from a study by Tekle and his colleagues, which proved that B7-H3 contributed to the metastatic capacity of melanoma cells by modulation of MMP-2 and signal transducer and activator of transcription 3 (Stat3) [22].

In conclusion, osteosarcoma originates from multiple cell types and has varied histological and biological characteristics. Its prognosis is poor and metastasis tends to already be recognized in more than half of all cases at the time of diagnosis. Our present findings indicate that the costimulatory molecule B7-H3 plays an important role in osteosarcoma progression, and might act as a negative regulator of T cells and help shielding tumors from immune surveillance. Strong B7-H3 expression was an independent prognostic factor for tumor-specific death in patients with osteosarcoma. Moreover, the expression of B7-H3 is correlated with the prevalence of early pulmonary metastases, which may facilitate the invasion of tumor cells in lymph nodes. Additional in-depth studies are required to understand the mechanism underlying the inhibitory function of B7-H3, and to further examine whether increased protein expression of B7-H3 in tumor could be useful in immunotherapeutic approaches.

## Supporting Information

Figure S1 **Representative immunostaining for B7-H3 expression in human melanoma tissue.** (A) positive control (B) negative control. ×200 magnification.
(TIF)
