# Supplementary figures and images for: B7-H3 is Overexpressed in Patients Suffering Osteosarcoma and Associated with Tumor Aggressiveness and Metastasis
Source: PLoS One. 2013 Aug 5;8(8):e70689. doi: 10.1371/journal.pone.0070689 (PMC3734259; doi:10.1371/journal.pone.0070689)

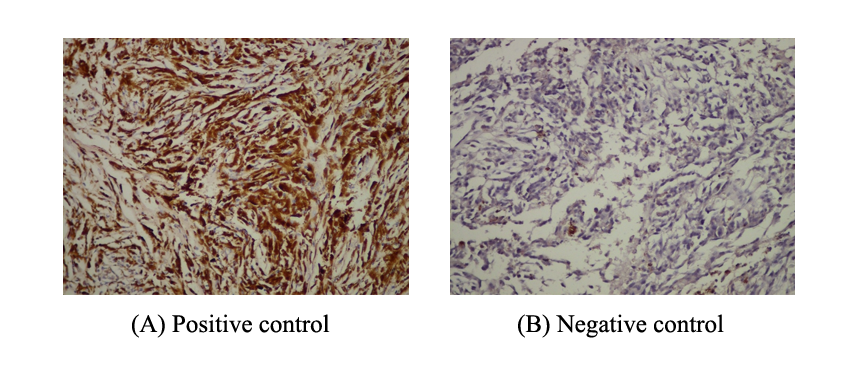

Supplement: Figure S1 — Representative immunostaining for B7-H3 expression in human melanoma tissue. (A) positive control (B) negative control. ×200 magnification. (TIF) [file pone.0070689.s001.tif]
